# Sonographic Measurement of Brainstem Through the Foramen Magnum in Premature Neonates Can Predict Neurodevelopment Outcome?

**DOI:** 10.3389/fneur.2021.770908

**Published:** 2021-12-24

**Authors:** Shyi-Jou Chen, Chih-Fen Hu, Chiung-Hsi Tien, Cheng-Yu Chen

**Affiliations:** ^1^National Defense Medical Center, Taipei, Taiwan; ^2^Department of Pediatrics, Tri-Service General Hospital, Taipei, Taiwan; ^3^Department of Radiology, School of Medicine, College of Medicine, Taipei Medical University, Taipei, Taiwan

**Keywords:** cranial ultrasound, trans-foramen-magnum high resolution ultrasound, preterm, brainstem, neurodevelopment

## Abstract

**Background:** To investigate whether serial morphometric measurements of the brainstem using high resolution trans-foramen-magnum ultrasound (US) in premature neonates correlate with neurological outcomes.

**Methods:** Serial brain ultrasound scans were performed in 36 consecutive preterm infants born at <34 weeks of gestation from birth until term-equivalent age. Two-dimensional brainstem measurements of the pons and medulla oblongata were compared with those in a cohort of 67 healthy full-term newborns. Neurologic assessment of the premature infants was assessed at 5 years of age.

**Results:** Of the 36 preterm infants born between 25 and 34 weeks of gestation, eight had significantly delayed growth profiles in both the pons and medulla and developed neurological sequelae by 5 years of age.

**Conclusions:** Morphometric measurements of the developing brainstem using high resolution trans-foramen-magnum ultrasound (US) may help predict neurological outcome in high-risk neonates, particularly in those who are born extremely premature.

## Introduction

The incidence of severe neurologic and developmental disabilities is high in children who were born as extremely preterm infants (1). Studies have shown that the poor cognitive and behavioral outcomes in many of these children are associated with reduced volumes of specific regions in the brain (2). Sonographic assessment is a valuable means of visualizing anatomical structures of the brain in fetuses and infants (3). Neonatal cranial sonography is traditionally performed through the anterior fontanelle; however, this approach does not offer good visualization of deep brain structures such as the brainstem and posterior fossa (4).

Studies have shown that better visualization of the neonatal midbrain and posterior fossa can be achieved when sonography is performed through alternative acoustic windows such as the posterior fontanelle, mastoid fontanelle, the temporal suture and the cisterna magnum (4–7).

Sonographic measurements of the cerebellum including transverse cerebellar diameter, the central vermian area, anterior-posterior distance and superior-inferior distance of the vermis have been assessed in preterm and term neonates with or without growth restriction (8–11). Furthermore, studies have shown that sonography performed via the foramen magnum is better at detecting abnormalities and anomalies in posterior fossa structures than scanning via the anterior fontanelle (12). To the best of our knowledge, however, no studies have evaluated whether this acoustic window is superior to the anterior fontanelle in measuring the medulla oblongata and the pons. In addition, no studies have performed ultrasound-based morphometric analyses of the developing medulla oblongata and pons in neonates. Therefore, in this study we conducted morphometric measurements of the medulla and pons via ultrasonography performed through the anterior fontanelle and foramen magnum to establish growth data of those two regions in healthy term neonates and to analyze the developmental changes of these regions in premature neonates with or without neurologic sequelae during the first 5 years of life.

## Materials and Methods

### Patients

In this observational cohort study, total of 67 healthy full-term neonates (gestational age: 38–41 weeks; 35 boys and 32 girls) were prospectively enrolled to determinate the morphometric reference values of the pons and medulla oblongata. In addition, we enrolled 36 premature neonates (gestational age: 25–34 weeks, 20 boys and 16 girls) to investigate whether the developmental curve of these regions could predict neurologic outcome. The 36 premature neonates were divided into two groups, namely a group of subjects with normal neurological development and a group of subjects with abnormal neurological consequences (five boys and three girls). Gestational age was determined according to the date of the mother's last menstrual period and/or the date on which sonography showed definitive pregnancy. All neonates were recruited with appropriate for gestational age and without small for gestational age nor fetal growth restriction. Apgar score are normal (≥7) at 1 and 5 min in term neonates after delivery and normal at 5 min in premature neonates. In addition, premature neonates who needed resuscitation (NRP) immediately after birth are excluded from this study. Infants were excluded from enrollment if there was clinical evidence of congenital infection or congenital malformation or if there was sonographic evidence of severe intraventricular hemorrhage (IVH) (grade III–IV). Infants were studied only after voluntary informed consent was obtained from the parents or guardians. The design of this study was approved by the institutional review board of Tri-Service General Hospital (IRB/REC-41948).

### Sonographic Evaluation

**Figure 1:**
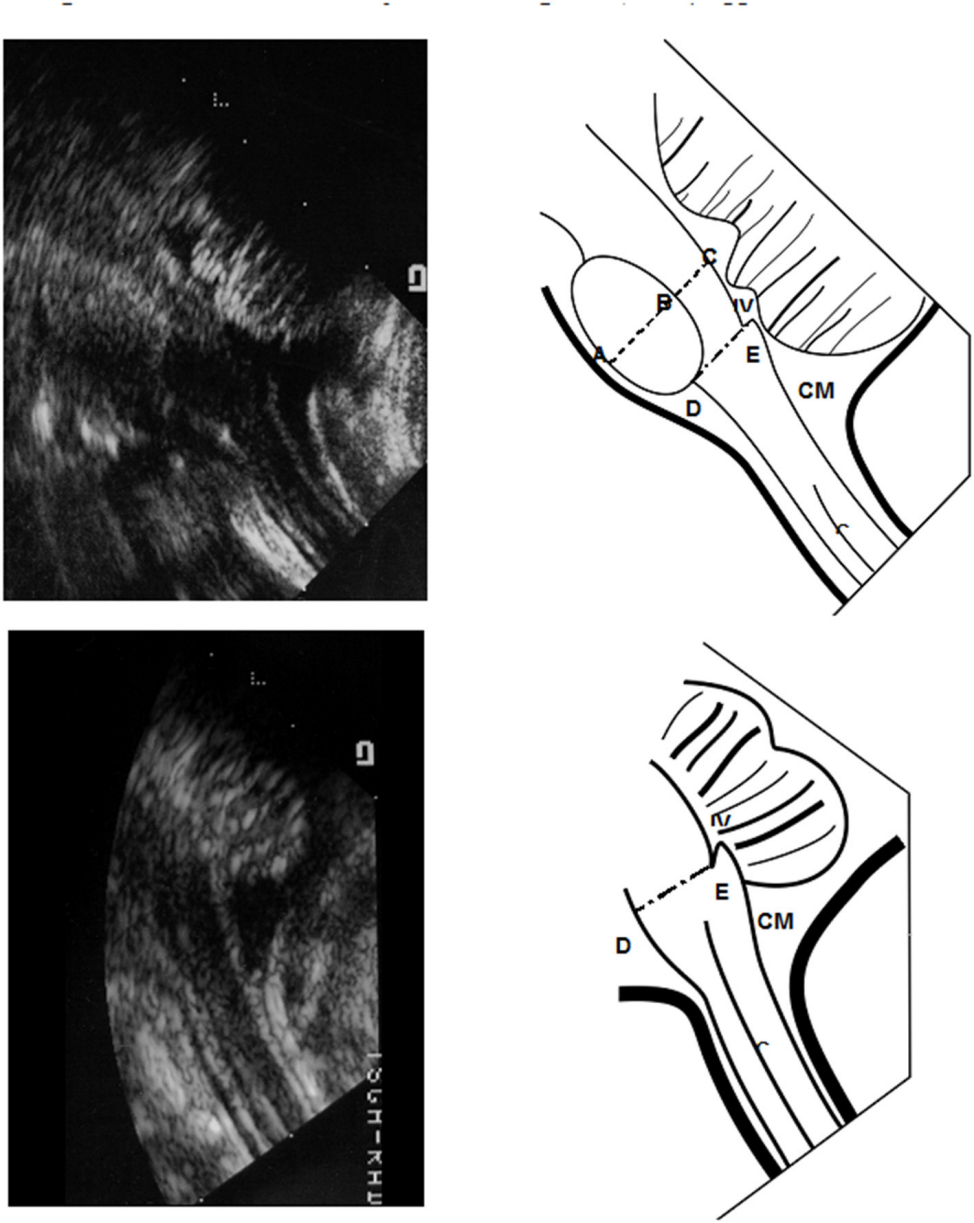
Morphometric reference values of pons and medulla oblongata from Sonograms taken via the trans-foramen magnum (TFM) approach. We demonstrate the landmark of diagrammatic sonography via convex transducer in Panel 1. The diameter of the midpons, area of the basic pons and diameter of the medulla oblongata were defined and measured as shown in Panel 1. The middle sagittal area of basic pons was quantified as the ovoid region. The transverse anteroposterior (AP) diameter of the midpons region **(A–C)** was calculated including the basic pons **(A,B)** and tegmentum **(B,C)**. The distance of the medulla oblongata was measured from **(D,E)**. IV, 4th ventricle; CM, cisterna magnum.

Cranial sonography was performed within 48 h after birth in normal full-term neonates and within the first 3 days of life in stable premature babies. Repeated scans were performed in premature infants once/2 weeks until discharge. All studies were performed with a high-resolution electronically focused real-time system (Acuson, Moutain View, CA) using a 7.5-MHz linear transducer. All scans were obtained in a midline sagittal view focused on the brainstem region through either the anterior fontanelle (AF) or foramen magnum (FM) as viewing acoustic windows. For performing trans-FM sonography, newborn was put in supine position with head gently bent forward similar to positioning lumbar puncture. We demonstrate the landmark of diagrammatic sonography via convex transducer in Figure 1.

The anterior-posterior (AP) diameters of the midpons (including the diameter of basis part and tegmentum part) and the oliver-shape area pons were measured in the mid-sagittal plane through either AF or FM acoustic windows (demonstrated in upper part of Figure 1). The area of pons was detected by following steps. First, we confirm midline sagittal cut of ultrasonic graph, and next we mark the top and bottom of Olivary-shape pons, and third, we extend the width of middle part of pons from top to down landmarks. Thus, we can circle the Olivary shape to cover overall and calculate the exact area of pons. We demonstrated in Supplementary Figure 1 accessed via AF window and Supplementary Figure 2 left figure via FM window. Meanwhile, midline sonography of the medulla oblongata was performed through the FM only (demonstrated in lower part of Figure 1). Vital signs including blood pressure, heart rate, body temperature and respiratory rate were monitored during each sonographic examination.

### Neurological Assessment

Neurodevelopmental outcome was assessed with Denver Developmental Screening Test II (DDST II) and determined at 5 years of age in all premature infants by pediatric neurologists.

### Statistical Analysis

Data were analyzed with SigmaPlot 11 statistical software (StatsDirect, Sale, United Kingdom). Calculations of average values were made for each item including both standard references (data collected either from of AF or from of FM) in healthy term children and the kinetic results of the developmental curves in premature babies with or without neurologic sequelae. Statistical results were significant at a level of *P* ≤ 0.05.

## Results

### Clinical Information

Of the 71 term newborns initially recruited, four dropped out of this study because of withdraw inform consent and one posterior fossa malformation with Dandy-Walker malformation. Of the 39 preterm newborns initially enrolled, 3 were excluded because of the development of grade IV IVH 2–3 weeks after birth (*n* = 2) or severe infection with bacteremia and meningitis during the period of investigation (*n* = 1). Therefore, 67 healthy full-term neonates (gestational age range, 38–41 weeks; 35 boys and 32 girls) were studied to determinate the morphometric reference values of the pons and medulla oblongata. Besides, 36 premature neonates (gestational age range, 25–34 weeks; 20 boys and 16 girls) (Table 1) were studied to investigate the developmental curve of these regions. The 36 premature neonates were divided into two groups, namely a group of 15 extremely premature neonates with gestational ages ranging from 25 to 30 weeks and a group of 21 preterm newborns with gestational ages ranging from 31 to 34 weeks (Table 1). At follow-up at 5 years of age, the incidence of neurologic sequelae was markedly higher among neonates with gestational ages of 25–30 weeks than among those with gestational ages of 31–34 weeks (Table 1). Among these eight cases with neurologic sequela, we list in the Supplementary Table, and the summary data of neurologic sequela are that five have developmental delay in motor function, four have ADHD, three have developmental delay in language, two have spastic diplegia, two have cognition disorder, one has monoplegia and one has epilepsy.

**Table 1:** Characteristics of preterm neonates with/without neurologic sequelae.

**Gestational age (Mean)**	**No. (M:F)**	**No. with neurologic sequelae (M:F)**
25–30 weeks	15 (9:6)	5 (3:2)
31–34 weeks	21 (11:10)	3 (2:1)
In Sum	36 (20:16)	8 (5:3)

#### Sonographic Measurements Taken Through the Foramen Magnum (FM) Window in Full-Term Infants

**Figure 2:**
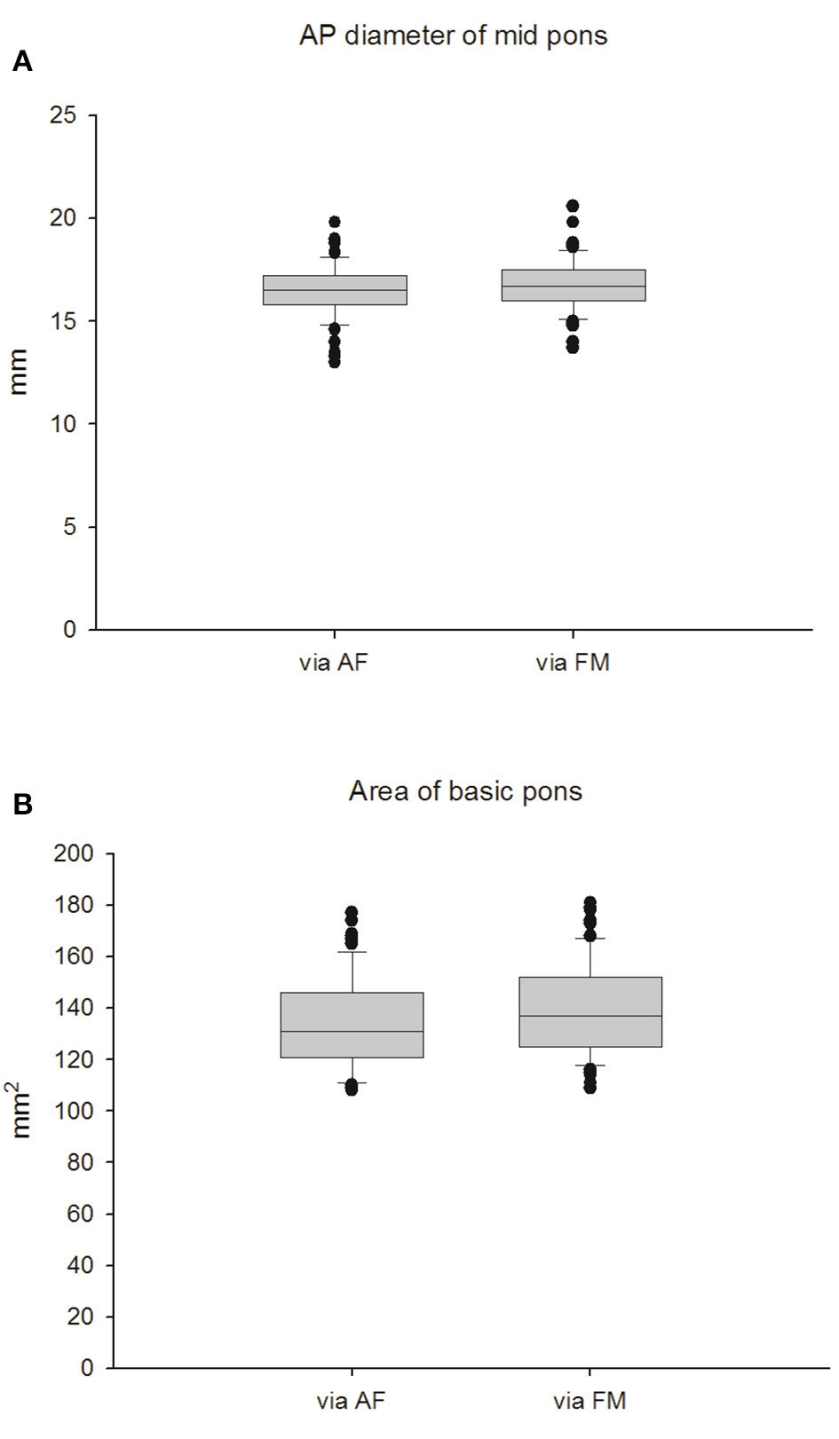
Measurement of the pons in full-term infants. **(A)** AP diameter of mid pons. **(B)** Area of basic pons.

Cranial ultrasound was performed either through the AF window or the FM window in healthy term newborns (Figure 1) and the data were summarized in Table 2. Through the AF approach, the mean transverse anteroposterior (AP) diameter of the midpons was 16.55 ± 1.36 mm (Figure 2A), that of the base of the pons was 10.74 ± 1.01 mm and that of the tegmentum was 5.81 ± 0.35 mm. Through the FM approach, the mean diameter of midpons was 16.71 ± 1.29 mm, that of the base of the pons was 11.01 ± 1.00 mm and that of the tegmentum was 5.70 ± 0.29 mm. As expected, there were no significant differences between AF and FM approaches in the measured values of the middle sagittal views (Figure 2B); however, there were significant differences in the measurements obtained through the AF. In addition, brainstem images obtained through the FM were clearer than those obtained through the AF. To further establish the reference values, we evaluated 2-dimensional images of the base of the pons (ovoid shape in Figure 1). The mean values obtained from the AF and FM approaches were 137.6 ± 17.5 mm^2^ and 139.7 ± 18.4 mm^2^, respectively (Figure 2). Similarly, there were no significant differences in the mean area of the base of the pons between the two acoustic widows. In addition, we assessed the AP diameters the medulla oblongata; however, the morphologic landmark of the medulla oblongata could only be demarcated on sonographic images taken through the FM window. The mean AP diameter of the medulla oblongata was 10.49 ± 0.114 mm through the FM window but unclear presentation of marker to detect AP diameter of the medulla oblongata through the AF window (Supplementary Figure 1). These results provided the fundamental reference values of healthy term neonates that could be used to evaluate the growth curves of the corresponding parameters in preterm newborns. Accordingly, we disclose that the FM window (Supplementary Figure 2) seem provide better interpretation to examine brainstem than AF widow especially in medulla area for neonatal cranial ultrasound.

**Table 2:** Measurement of pons and medulla oblongata in full-term infants.

**Region of evaluation**	**Trans-foramen magnum**
AP diameter of middle pons	16.71 + 1.29 mm
Basis part	11.01 ± 1.00 mm
Tegmentum part	5.7 ± 0.29 mm
Area of pons	139.8 ± 2.24 mm^2^
AP diameter of medulla oblongata	10.49 ± 0.11 mm

#### Comparison of Sonograms of the Pons Taken via the AF Approach With Those Taken via the FM Approach in Premature Infants

**Figure 3:**
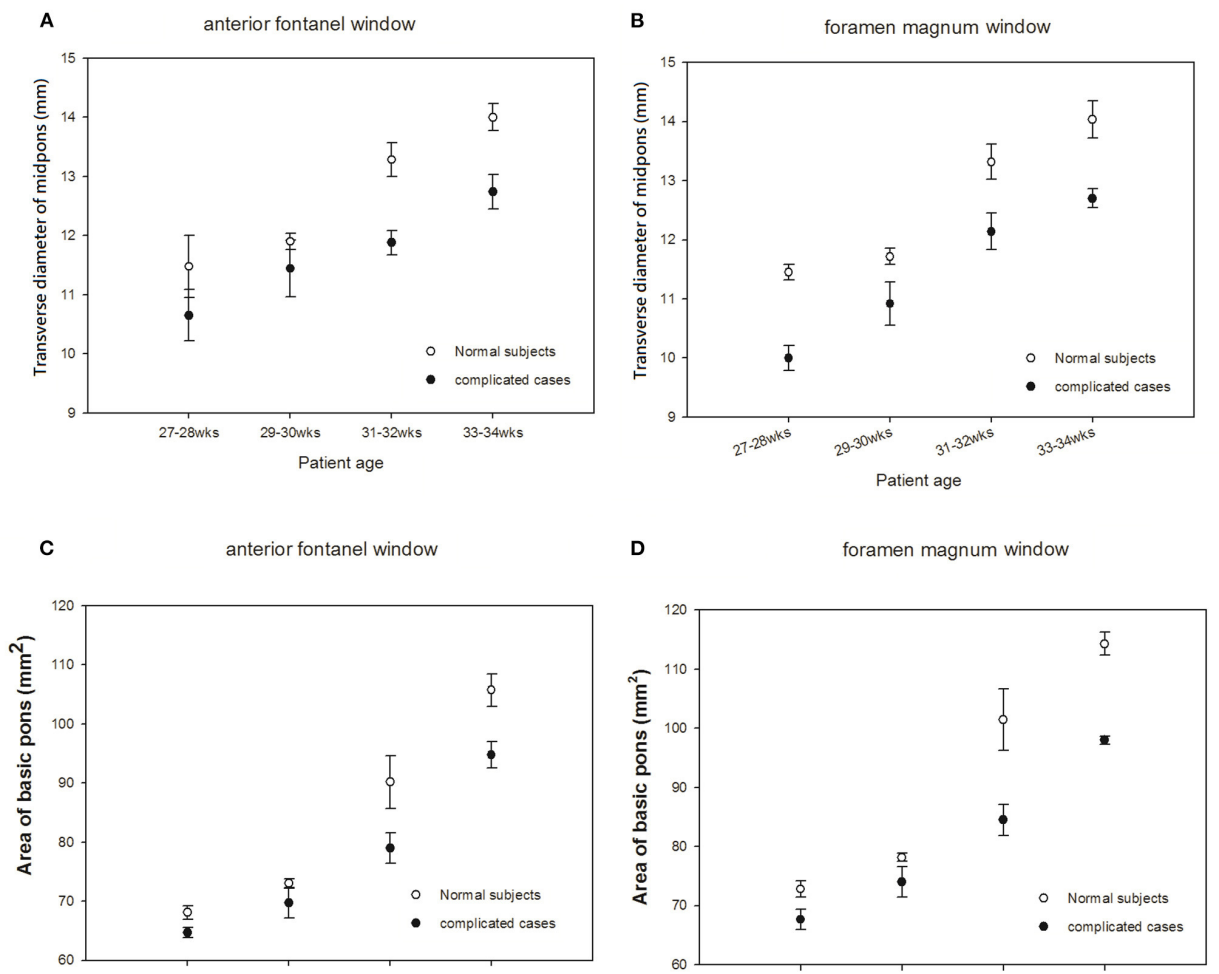
Comparisons of sonograms of the pons in neonates with normal development with those in neonates with neurologic sequelae. **(A,C)** anterior fontanel window. **(B,D)** foramen magnum window.

**Figure 4:**
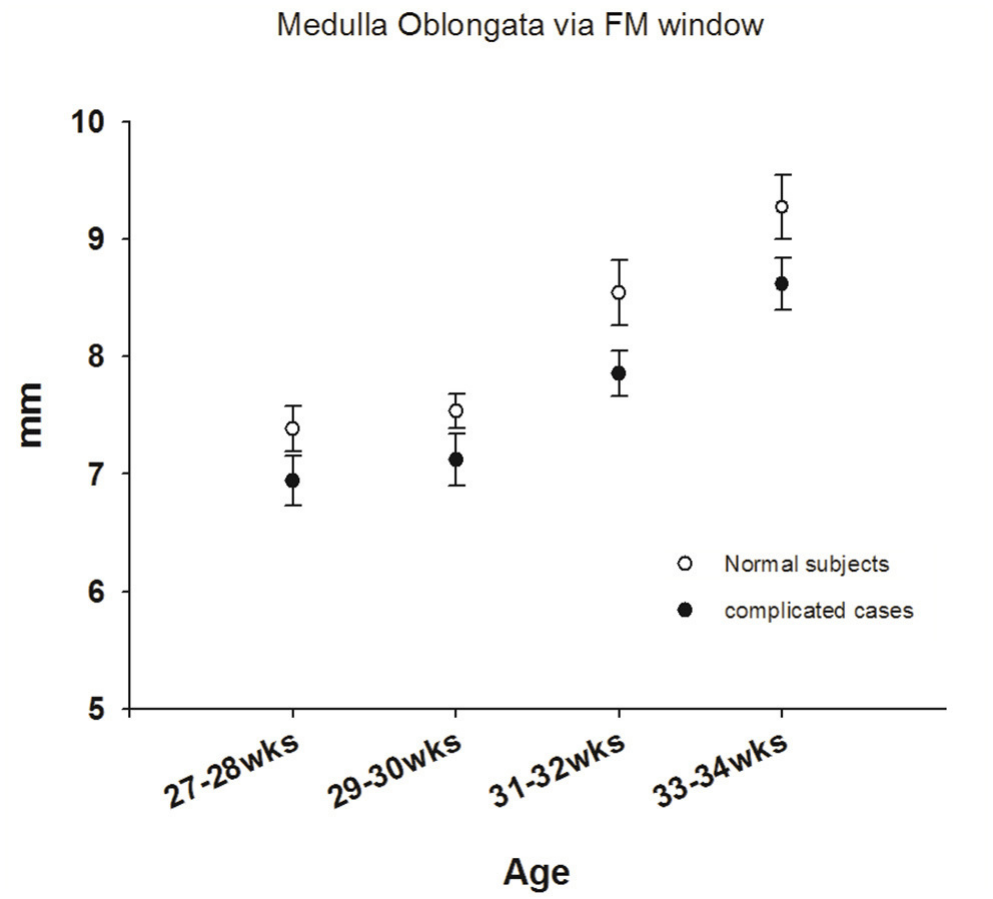
Comparisons of sonograms of the medulla oblongata in neonates with normal development with those in neonates with neurologic sequelae.

To investigate the development of the pons, we measured the AP diameter of the pons (Figures 3A,B) and the area of base of the pons in premature neonates (Figures 3C,D). Due to the diverse gestational age at birth of premature cases when they were enrolled, thus data shown in Figures 3, 4, ultrasonic measurements were taken at their corresponding gestational ages. Two cases born at 25 weeks of gestational age (GA) due to increasing risk of hypothermia and/or desaturation when executed brain sonography via the window of foramen magnum at 25 or at 26 weeks of GA. So, we started collecting data after 27 weeks of corresponding GA in this study. There were no significant differences in measured data between those obtained via the AF approach and data obtained via the FM approach. Interestingly, we found that the growth velocities in AP diameter and area of the pons differed between premature infants with normal development and those with neurologic sequelae at five years of age. In premature infants with normal development, the rate of growth of the two pons parameters was rather slow before the age of 30 weeks followed by a rapid increase in development during the period of 31–32 weeks' GA. In preterm infants who developed neurologic problems, however, there was steady slow growth of the pons throughout the measurement period (Figures 3A–D). This finding suggests that the development of the pons can be used to predict subsequent neurologic sequelae.

#### Assessment of Development of the Medulla Oblongata in Preterm Newborns

The medulla oblongata is another pivotal structure comprising the brainstem. To address the correlation between neurologic sequelae and age-dependent neurologic maturation, we investigated medulla oblongata in all term and preterm newborns. Disappointingly, images of the medulla oblongata obtained through the AF window were too obscure to define the landmark; therefore, we only analyzed images of the medulla oblongata obtained via the FM approach. The mean diameter of the medulla oblongata in healthy term newborns was 10.5 ± 0.93 mm (Max: 12.2 mm; Min: 8.3 mm) via the FM approach (Table 2). We observed an initially significant slow growth curve of the medulla oblongata in premature neonates born before 32 weeks of gestational age who developed neurologic sequelae. Similarly, we observed an increasing rapid growth rate of the medulla oblongata in all preterm infants born at 31–32 weeks' GA (Figure 4), a finding similar to that reported by Amin et al. (13). However, there was no significant difference in size of the medulla between the two groups during the period of investigation, which implies that the medulla oblongata and pons mature at different rates.

## Discussion

The structures in the posterior cranial fossa in neonates and infants can be visualized ultrasonographically via the anterior fontanelle; however, posterior fontanelle ultrasound allows for better comprehensive delineation of occipital lobe structure (14). Previously, Ichiyama and Hayashi evaluated the morphological abnormalities in the posterior cranial fossa in neonates and in 1–6 month-old infants by measuring the diameters of the cerebellar vermis, pons, fourth ventricle and cisterna vermis in the midsagittal plane and this study demonstrated that the structures of the posterior fossa can be apparently pictured until 6 months after birth by means of ultrasonography through the AF (6). However, the authors did not provide longitudinal follow-up data in neonatal cases.

Posterior fontanelle imaging demonstrates subtle differences in echogenicity between clots and the choroid glomus and can depict clots extending into the occipital and temporal horns. This scanning method also allows for visualization of subarachnoid blood and clots obstructing the outlet of the fourth ventricle (15). Mastoid fontanelle imaging has been used to detect hemorrhage involving the brainstem, cerebellum, and subarachnoid cisterns. However, few studies have evaluated the risk factors associated with the development of these ultrasound abnormalities or the neurobehavioral outcome of infants with said abnormalities (16). In our future research, in the metric study of the brainstem via FM window we will use a multifrequency micro-convex probe (5–8 MHZ), which allows the operator to improve the image solution and provide more depth for the assessment of anomalies and biometrics of the posterior fossa structures, as shown by Brennan and Taylor (17). Thus, we will apply this multifrequency Micro-Convex probe (5–8 MHZ) for our advanced research in the measurement of brainstem via FM window in the future. In this study, we measured the diameters of the pons and medulla oblongata using ultrasound performed via the foramen magnum (FM) to try to establish the normal parameters in brainstem development in term and premature neonates. Behnke et al. reported that infants determined to be at higher perinatal risk were more likely to have cranial ultrasound abnormalities identified at birth and that infants with cranial ultrasound abnormalities identified at birth would perform more poorly on standardized tests of newborn neurobehavioral function (18). The results of our investigation show that abnormal neurobehavioral outcome is associated with significant delayed growth velocity of brainstem in preterm newborns. Reduction in volumetric measurements of the brain have been shown to be associated with poor cognitive and behavior outcome, particularly in children who were born extremely premature (19). We disclosed these eight cases with neurologic sequela all have RDS and high incidence of PVL grade I or II, and the most severe motor deficit is case No. 2 with moderate spastic diplegia who also has IVH grade II and ROP grade III implying the possible correlation with the severity of neurologic sequela. However, we cannot make a conclusion for these limited data. Thompson et al. used magnetic resonance imaging to study perinatal risk factors that alter regional brain structure in preterm infants and found that some regions of cortical gray matter and unmyelinated white matter were clearly reduced in preterm infants (20). Cebeci et al. demonstrated the influential result of proton magnetic resonance spectroscopy (H-MRS) and N-acetylaspartate (NAA) on the prediction of brain development and neurologic outcome (21), but MRI/MRS is not easily to apply in NICU regularly.

To the best of our knowledge, however, few studies have investigated the potential effects of volume of brain regions on neurobehavioral outcome in children who were born extremely premature. Nevertheless, some limitations are emerged in this study. Restrictedly, we enrolled premature cases with normal Apgar scare at 5 min without resuscitation after birth. Due to few cases of extreme prematurity are enrolled, we cannot confirm the correlation of Apgar score and abnormal neurologic outcome of premature group in this study. In addition, we cannot detect apparent vision of medulla oblongata via traditional AF window to evaluate the exact size of the width of medulla oblongata as comparisons of detection via FM window. For ergonomic limitation, the linear scan transducer is not easily to handle when perform sonography through FM window in extreme premature babies younger than 27 corresponding gestational ages. Moreover, cases born at 25 GA are under high risk of hypothermia and/or desaturation during this procedure via FM window, leading us to collect data since the corresponding GA of 27 weeks. Due to limited case numbers of premature groups were enrolled leading to difficulty of statistical analysis. Besides, for the data merged at corresponding GA, we only showed the growth tendency but not the individual growth curve of each case precisely. As well, we did not report comprehensively for Apgar score, classification of weight in comparisons with biometric measurements of brainstem and neurologic development. Thus, a well-designed and extend prospective study should be conducted to improve these restrictions, to provide more informations.

In summary, in this study, we observed a rapid growth curve beginning at 31 weeks of GA in well-developed preterm newborns but only a steady slow growth curve in these measurements of the pons in preterm infants who subsequently developed neurologic problems (Figures 3A–D). Our findings suggest that the degree of brainstem maturation can predict neurological sequelae in high risk neonates. Sonography performed via the foramen magnum (FM) provides good visualization of the medulla oblongata. Thus, we also observed a similar trend in development of the medulla oblongata as measured through the FM window. Nevertheless, more data and a longer observation period are necessary to confirm the relationship among gestational age, growth curves of brainstem and to be a predictive parameter for evaluation of neurobehavioral development in premature neonates.

## Data Availability Statement

The raw data supporting the conclusions of this article will be made available by the authors, without undue reservation.

## Ethics Statement

The studies involving human participants were reviewed and approved by Institutional Review Board, Tri-service General Hospital. Written informed consent to participate in this study was provided by the participants' legal guardian/next of kin.

## Author Contributions

S-JC performed ultrasonography, data collection, data analysis, and writing of the manuscript. C-FH and C-HT follow up the cases and clinical evaluation a part and assist the collection of data. C-YC designed the study, defined the landmark of brainstem ultrasonography, and guide ultrasonography as well as instructed performing ultrasonography. All authors contributed to the article and approved the submitted version.

## Funding

This work was supported by grants from the MOST-109-2314B-016-023, TSGH-C01-109014, 801GB110034, CTH-101-2C01, and TSGH-C-110031 to S-JC.

## Conflict of Interest

The authors declare that the research was conducted in the absence of any commercial or financial relationships that could be construed as a potential conflict of interest.

## Publisher's Note

All claims expressed in this article are solely those of the authors and do not necessarily represent those of their affiliated organizations, or those of the publisher, the editors and the reviewers. Any product that may be evaluated in this article, or claim that may be made by its manufacturer, is not guaranteed or endorsed by the publisher.

## References

[1] Wood NS Marlow N Costeloe K Gibson AT Wilkinson AR. Neurologic and developmental disability after extremely preterm birth. EPICure Study Group. N Engl J Med. (2000) 343:378–84. 10.1056/NEJM200008103430601 10933736

[2] Bhutta AT Anand KJ. Abnormal cognition and behavior in preterm neonates linked to smaller brain volumes. Trends Neurosci. (2001) 24:129–30. 10.1016/s0166-2236(00)01747-1 11182440

[3] Pigadas A Thompson JR Grube GL. Normal infant brain anatomy: correlated real-time sonograms and brain specimens. Am J Roentgenol. (1981) 137:815–20. 10.2214/ajr.137.4.815 6974979

[4] Hashimoto K Takeuchi Y Takashima S Takeshita K. Morphometric evaluation of neonatal brainstem development by means of the ultrasonographic method. Brain Dev. (1994) 16:209–12. 10.1016/0387-7604(94)90071-X 7943605

[5] Helmke K Winkler P Kock C. Sonographic examination of the brain stem area in infants. An echographic and anatomic analysis. Pediatr Radiol. (1987) 17:1–6. 10.1007/BF02386584 3822577

[6] Ichiyama T Hayashi T. Ultrasonic measurements of the posterior cranial fossa structures in neonates and infants. Eur J Pediatr. (1991) 150:719–21. 10.1007/BF01958763 1915484

[7] Buckley KM Taylor GA Estroff JA Barnewolt CE Share JC Paltiel HJ. Use of the mastoid fontanelle for improved sonographic visualization of the neonatal midbrain and posterior fossa. Am J Roentgenol. (1997) 168:1021–5. 10.2214/ajr.168.4.9124108 9124108

[8] Birnholz JC. Newborn cerebellar size. Pediatrics. (1982) 70:284–7. 7099797

[9] Huang CC Liu CC. The differences in growth of cerebellar vermis between appropriate-for-gestational-age and small-for-gestational-age newborns. Early Hum Dev. (1993) 33:9–19. 10.1016/0378-3782(93)90169-U 8319557

[10] Makhoul IR Goldstein I Epelman M Tamir A Reece EA Sujov P. Neonatal transverse cerebellar diameter in normal and growth-restricted infants. J Matern Fetal Med. (2000) 9:155–60. 10.3109/14767050009020520 10914622

[11] Imamoglu EY Gursoy T Ovali F Hayran M Karatekin G. Nomograms of cerebellar vermis height and transverse cerebellar diameter in appropriate-for-gestational-age neonates. Early Hum Dev. (2013) 89:919–23. 10.1016/j.earlhumdev.2013.10.001 24183100

[12] Sudakoff GS Montazemi M Rifkin MD. The foramen magnum: the underutilized acoustic window to the posterior fossa. J Ultrasound Med. (1993) 12:205–10. 10.7863/jum.1993.12.4.205 8497026

[13] Amin SB Merle KS Orlando MS Dalzell LE Guillet R. Brainstem maturation in premature infants as a function of enteral feeding type. Pediatrics. (2000) 106:318–22. 10.1542/peds.106.2.318 10920158

[14] Anderson NG Hay R Hutchings M Whitehead M Darlow B. Posterior fontanelle cranial ultrasound: anatomic and sonographic correlation. Early Hum Dev. (1995) 42:141–52. 10.1016/0378-3782(95)01648-M 7588159

[15] Cramer BC Jequier S O'Gorman AM. Sonography of the neonatal craniocervical junction. Am J Roentgenol. (1986) 147:133–9. 10.2214/ajr.147.1.133 3521235

[16] Salvo DNDI. New view of the neonatal brain: clinical utility of supplemental neurologic US imaging windows. Radiographics. (2001) 21:943–55. 10.1148/radiographics.21.4.g01jl14943 11452069

[17] Brennan CM Taylor GA. Sonographic imaging of the posterior fossa utilizing the foramen magnum. Pediatr Radiol. (2010) 40:1411–6. 10.1007/s00247-010-1635-5 20336286

[18] Behnke M Eyler FD Garvan CW Tenholder MJ Wobie K Woods NS. Cranial ultrasound abnormalities identified at birth: their relationship to perinatal risk and neurobehavioral outcome. Pediatrics. (1999) 103:e41. 10.1542/peds.103.4.e41 10103333

[19] Peterson BS Vohr B Staib LH Cannistraci CJ Dolberg A Schneider KC. Regional brain volume abnormalities and long-term cognitive outcome in preterm infants. JAMA. (2000) 284:1939–47. 10.1001/jama.284.15.1939 11035890

[20] Thompson DK Warfield SK Carlin JB Pavlovic M Wang HX Bear M. Perinatal risk factors altering regional brain structure in the preterm infant. Brain. (2007) 130:667–77. 10.1093/brain/awl277 17008333

[21] Cebeci B Alderliesten T Wijnen JP van der Aa NE Benders M Vries LSde. Brain proton magnetic resonance spectroscopy and neurodevelopment after preterm birth: a systematic review. Pediatr Res. (2021). 10.1038/s41390-021-01539-x 33953356

