# Supplementary material for: Sonographic Measurement of Brainstem Through the Foramen Magnum in Premature Neonates Can Predict Neurodevelopment Outcome?
Source: Front Neurol. 2021 Dec 24;12:770908. doi: 10.3389/fneur.2021.770908 (PMC8739975; doi:10.3389/fneur.2021.770908)
Supplement: Supplementary file 1 [file Data_sheet_1.docx]

**Supplementary Table.** Neurologic sequela in eight premature cases

| **No** | **Gender** | **GA** | **Complication in neonatal stage** | **Neurologic outcome at 5 Y/O** |
| --- | --- | --- | --- | --- |
| 1 | M | 25 2/7 Wks. | RDS, BPD, PVL bil. Grade II | Developmental delay in language and motor, mild spastic diplegia |
| 2 | M | 27 3/7 wks. | RDS, BPD, PVL and IVH grade II, ROP stage III, PDA S/P ligation | Developmental delay in language， cognition and motor, moderate spastic diplegia |
| 3 | F | 29 5/7 Wks. | RDS, Apnea, IVH, Rt grade I | Developmental delay in fine motor |
| 4 | F | 29 6/7 Wks. | RDS, PDA, Apnea of prematurity, neonatal sepsis | Developmental delay in fine motor and ADHD |
| 5 | M | 30 5/7 Wks. | RDS, R/O perinatal infection, R/O neonatal seizure | Epilepsy, ADHD |
| 6 | M | 32 Wks. | RDS, Twin B, Hypotension | Developmental delay in language and cognition, and ADHD |
| 7 | F | 33 1/7 Wks. | RDS, PVL Lt., grade II | Developmental delay in and gross motor, mild Rt monoplegia |
| 8 | F | 34 Wks. | Mild, PVL, grade 1 | ADHD |

Abbreviation: M: male, F: female, GA: gestational age, Y/O: years old, Wks.: weeks, RDS: respiratory distress syndrome; BPD: bronchopulmonary dysplasia, PVL: periventricular leukomalacia; IVH: intraventricular hemorrhage, ROP: retinopathy of prematurity, ADHD: attention deficit and hyperactivity


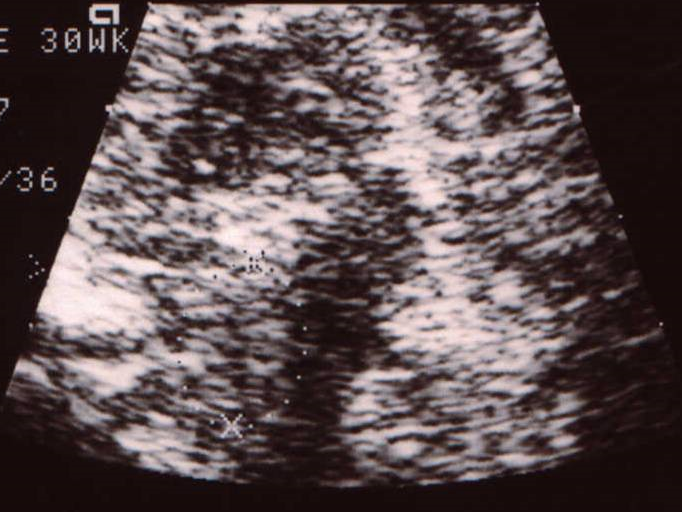


**Supplementary Figure S1.** Sonography of Oliva shape of pons accessed via Anterior Fontanel window in neonate of 37 gestational age with limitation to access the apparent image of medulla.


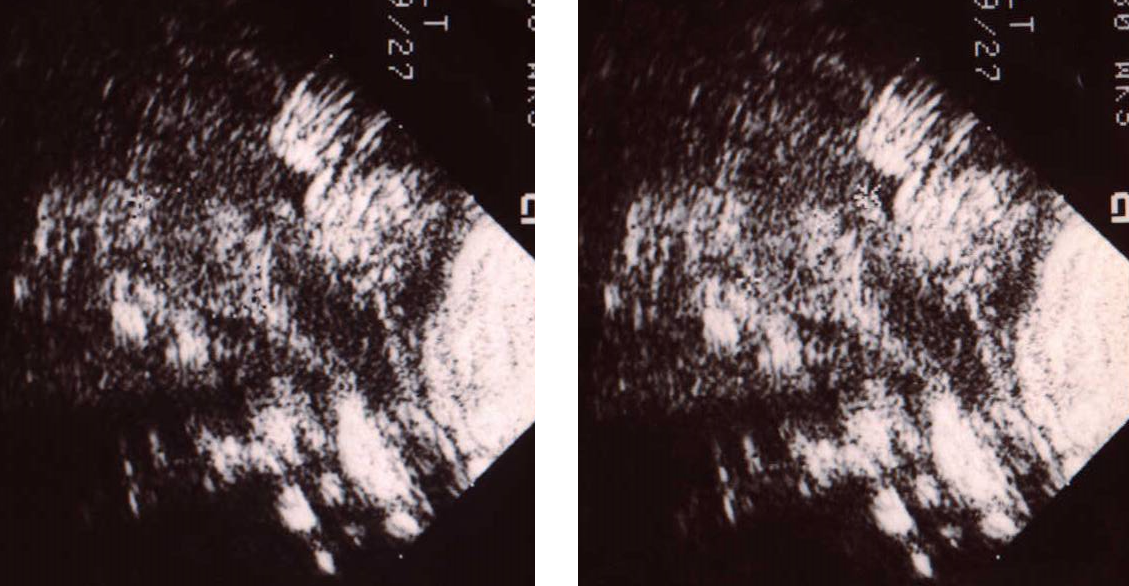


**Supplementary Figure S2.** Well demarcation of Oliva shape of pons sonography accessed via Foramen Magnum window and obvious detection of medulla image.
